# Acute Physical Activity Enhances Executive Functions in Children with ADHD

**DOI:** 10.1038/s41598-018-30067-8

**Published:** 2018-08-17

**Authors:** Valentin Benzing, Yu-Kai Chang, Mirko Schmidt

**Affiliations:** 1 Institute of Sport Science, University of Bern, Bern, Switzerland; 2 Department of Physical Education, National Taiwan Normal University, Taipei, Taiwan

## Abstract

Acute physical activity of moderate to vigorous intensity has been shown to improve cognitive functions in children. However, the empirical evidence associated with Attention Deficit Hyperactivity Disorder (ADHD) in children is still limited, in particular regarding which specific cognitive functions benefit. This study investigated the effects of an acute bout of physical activity on multiple aspects of executive functions (inhibition, switching, and visual working memory) in children with ADHD. Forty-six children (8–12 years old; 82.6% boys) were randomly assigned to either 15 minutes of acute exergaming (physical activity of moderate intensity) or to a control condition (sedentary). Executive function performance in inhibition, switching and visual working memory were assessed before and after each condition, using a modified version of both the Flanker and the Color Span Backwards Task. The results revealed that participants in the exergaming group performed significantly faster than those in the control group in terms of both inhibition and switching, but there was no significant difference in the accuracy of the two tasks nor in visual working memory performance. These findings suggest that acute physical activity utilizing exergaming has the potential to improve specific aspects of executive functions (reaction times in inhibition and switching) in children with ADHD.

## Introduction

Attention Deficit Hyperactivity Disorder (ADHD) is recognized as a highly prevalent disorder (3–7%) in childhood and adolescence^1,2^. The male-to-female ratio in community-based samples is approximately 3:1^1^, whereas it ranges between 5:1 and 9:1 in clinical samples^3^. The primary symptoms of ADHD are inattention, disorganisation, and hyperactivity-impulsivity^4^. These symptoms are frequently observed at pre-school age and often persist into adulthood^5^. Children with ADHD display an increased risk of suffering from social, long-term academic, and work-related impairments^6^, creating a large social burden^7^.

ADHD is linked to lower Executive Function (EF) performance and motor deficits^8–10^. EFs are defined as the higher-order cognitive functions that modulate fundamental cognitive processes and are therefore required for goal-oriented, adaptive and flexible behavior^11,12^. EFs are thought to be comprised of three core processes^13^: (1) *inhibition*, which includes inhibiting predominant responses and controlling attention; (2) *switching*, which includes switching between tasks or mental sets; and (3) *working memory*, which includes retaining and processing information. Deficiency of the aforementioned EFs is seen as one prevailing explanation for the development of ADHD^10^, with previous studies suggesting that pronounced ADHD symptomatology is associated with poor EF performance^14–17^.

A predominant model for the underlying mechanisms of ADHD describes dysfunction of the prefrontal-striatal circuitry^18^, where abnormal prefrontal-striatal-related cortices, such as the prefrontal cortex or basal ganglia, could underpin EF impairment in ADHD^18,19^. For example, children with ADHD display pronounced hypoactivation during cognitive task performance. This hypoactivation has been detected in systems involved in the frontoparietal and the ventral attentional network, both of which are supposed to be linked to EFs and attention^20,21^. Another proposed mechanism is associated with disrupted catecholamine neurotransmission, meaning that individuals with ADHD have decreased levels of dopamine and norepinephrine in brain networks that are connected to EFs and attention, resulting in decreased cognitive performance^22^.

Although the empirical evidence in children is limited, it seems that single bouts of exercise (also known as acute exercise or acute physical activity) have a positive impact on neurotransmission and brain functioning^23,24^. Research exists showing that acute exercise seems to trigger catecholamine neurotransmission^24,25^, which in turn is thought to beneficially affect EFs by increasing the allocation of attention^26^. Further evidence can be found in the fact that acute exercise seems to lead to modulated event-related potentials (ERPs). These modulations include increased P3 amplitudes and shorter latencies, which, alongside producing benefits for cognitive performance, can also be interpreted as an increase in attentional resources^27,28^. Interestingly, it has recently been shown in adults that acute exercise can increase functional connectivity in a variety of large-scale networks which are thought to be related to EFs and attention, such as the frontoparietal and the ventral attentional network^29^. Moreover, Weng *et al*.^29^ have proposed that the benefits elicited by acute exercise might stem from an exercise-induced release of catecholamines, such as dopamine and norepinephrine^24,30^. The association between acute exercise, functional connectivity, and catecholamines may be particularly relevant for children with ADHD, since these effects appear to be localized in brain regions that are also involved in the pathophysiology of the disorder.

A substantial body of evidence supports the association between exercise and cognitive function in healthy children^31–35^. However, a recent meta-analysis found acute physical activity to have positive effects only on inhibition, but not on switching and working memory^32^. Few studies have investigated the effects of acute physical activity on multiple EFs in children with ADHD. The majority of these studies investigated the effects of exercise on inhibition, consistently finding beneficial effects^36–40^. To date, three studies have looked into the effects on switching^36,39,41^. Two of these studies found positive effects on switching indexes, whereas one study^39^ found no significant differences. Lastly, only two studies have examined the effects of acute physical activity on working memory in children with ADHD^41,42^, both revealing no significant effects. Taking into account the consistent beneficial effects of acute physical activity observed on inhibition, further research should include all three core EFs in children with ADHD, especially considering that recent studies have supported the selective benefits of acute physical activity on EFs in healthy schoolchildren^43^.

As previously mentioned, acute exercise is positively linked to cognitive functions in healthy children. However, it seems that not all core EFs benefit equally from it^32^. In children with ADHD, research into which specific cognitive functions benefit is limited. The existing studies have mainly investigated inhibition, with few studies focusing on switching and working memory aspects of EFs. No study has investigated the effects on the three core EFs in children with ADHD in a single study. The purpose of this study was therefore to advance our current understanding by investigating whether acute physical activity selectively affects the three core EFs (inhibition, switching and working memory) in children with ADHD. We hypothesized that acute physical activity would elicit significant benefits to inhibition and switching performance in children with ADHD; however, considering the limited evidence on working memory, no directional hypothesis was formed for working memory.

## Materials and Methods

### Participants

51 participants between the ages of 8 and 12 years were recruited through an association for parents and caregivers of children with ADHD. The participants had been diagnosed with ADHD based upon the International Classification of Disease (ICD-10^44^) by a medical professional. However, participants with neurological disorders, Tourette syndrome or epileptic disorders were excluded.

The parents of the eligible participants completed assessments providing demographic information, including pubertal status, socioeconomic status, physical activity behavior, and data on the ADHD diagnosis. Pubertal status was assessed using the German version of the *Pubertal Developmental Scale*^45^. For each gender, it consists of three questions (e.g. for boys, “Have you noticed a deepening of your son’s voice?”). Responses had to be given on a 4-point Likert scale, scoring 1–4 points (e.g., not yet started; barely started; definitely started; seems complete). The respective sum constitutes the puberty index (3–12 points). An acceptable reliability and validity has been demonstrated^45^. The socioeconomic status was assessed using the *Family Affluence Scale II*^46^. This consists of four questions regarding the family (e.g. whether their child has its own bedroom, the number of family-owned cars etc.). The response format varies from item to item, and points are given for a higher number, for example the number of family-owned cars. The sum of the four items ranges between 0 and 9 and constitutes the prosperity index. An acceptable reliability and validity has been demonstrated^46^. Physical activity behavior was assessed using the *Physical Activity, Exercise, and Sport Questionnaire*^47^. Parents had to indicate the frequency and duration of up to three types of exercise that their children regularly engage in, resulting in an average number of minutes per week. Acceptable psychometric properties have been demonstrated^47^. Lastly, ADHD symptoms were assessed using *Conners-3*^48^. This questionnaire, whose reliability, validity and internal consistency are well-established^49^, consists of 108 items (rated on a 4-point Likert scale). For the present study, *t*-scores on DSM-IV-TR symptom scales (hyperactivity, inattentiveness and combined) and the global index *t*-score were used to measure severity of ADHD. *T*-scores of 60–64 are considered borderline; *t*-scores of 65–69 are considered clinically elevated; *t*-scores of 70 and above are considered clinically elevated. The participants’ background demographics are presented in Table 1.

**Table 1:** Participants’ demographics, comparing Exergaming and Control groups (means and standard deviations).

	Exergaming (*n* = 24)	Control (*n* = 22)	*p*
	*M* (*SD*)	*M* (*SD*)	
Age	10.46 (1.35)	10.50 (1.41)	0.919
Gender (% male)	83.3%	81.8%	
Height	144.52 (10.44)	144.66 (10.20)	0.771
Weight	36.67 (7.80)	40.70 (15.55)	0.223
Pubertal developmental status	3.80 (1.11)	3.50 (0.79)	0.594
Socioeconomic status	6.56 (1.21)	6.33 (1.49)	0.558
Physical activity behavior (minutes/week)	150.64 (144.55)	149.43 (140.19)	0.977
ADHD Diagnosis	100%	100%	
DSM-IV Symptoms (Conners-3)			
Hyperactivity	62.77 (4.91)	62.85 (6.18)	0.373
Inattentiveness	64.58 (4.63)	62.67 (7.36)	0.648
Combined	65.08 (4.19)	63.43 (6.38)	0.453
Global index	66.25 (4.60)	65.24 (7.04)	0.485
Medication	79.2%	77.3%	

46 participants successfully completed the study; five participants (two in control; three in experimental groups) were unable to attend the second cognitive assessment for personal reasons. As shown in Table 1, participants were on average 10.48 years old (*SD* = 1.38) and 82.6% of them were boys. The participants’ pubertal and socioeconomic status was about average and the severity of their ADHD symptoms can be considered borderline to clinically elevated with regard to the global index. Most participants were regular users of stimulant medication.

The cantonal ethics committee approved the study protocol, which adhered to the latest version of the declaration of Helsinki. The trial was registered at the German Clinical Trials Register (DRKS) on March 14, 2016. The registration number is DRKS00010171. The legal guardians of all participants provided written informed consent.

### Design and procedure

In a between-subjects design, participants were randomly assigned either to an *Exergaming* or a *Control* group (this assignment being concealed from both the researchers and participants). The study was conducted in the canton of Bern, Switzerland. Both the acute intervention and the assessments took place in a quiet room at the respective family’s home, to make participating in the study as convenient as possible. Testing was scheduled between 1 p.m. and 4 p.m. in both groups and did not differ between the two groups (*p* > 0.05). Participants were blinded with regard to the study aims and conditions. After signing the informed consent and assessing demographic information, the participants were fitted with heart rate monitoring equipment. EF performance was assessed prior to (pre-test) and following (post-test) the acute intervention. Each acute intervention (i.e., *Exergaming* or *Control* condition) lasted approximately 15 minutes, including a short break of approximately one minute, during which the participants provided pleasure and arousal ratings. Immediately after the activity (post-test), perceived physical exertion, cognitive engagement and enjoyment of the activity were measured. After completing the experimental procedures, the participants received a small gift, and the purpose of the experiment was explained to the legal guardians.

### Acute intervention

In the *Exergaming* condition, participants were asked to play an exergame (portmanteau for exercising and gaming) named “Shape Up” (Ubisoft, Montreal, Canada), which has been shown to be physically (i.e. moderate to vigorous intensities) and cognitively challenging^50^.

The *Exergaming* condition was conducted using the XBOX Kinect (Microsoft, Redmond, WA). This is a game console including a motion-sensing input device. Users control and interact with the console through their body movements. The user is projected directly into the virtual reality on the screen by integrated cameras. Within “Shape Up”, users completed the “Beatmaster Training Quest”, which consists of six different exercises: (1) In “Waterfall Jump”, the player stands on the edge of a waterfall and has to jump onto oncoming pieces of wood in order not to fall down. These pieces of wood are of different sizes and have footprints on them (left or right foot only, or both feet together). The frequency and order of the footprints vary. (2) In “Stunt Run”, the player has to sprint on top of a moving train and react as quickly as possible to upcoming obstacles, which force the player to do sidesteps, jump over or duck down. (3) In “Derby Skate”, the player has to imitate and learn new sequences of movements, comparable to aerobics. (4) In “Squat Me To The Moon”, the player has to perform a series of deep and fast squats in order to become the first person on the moon. (5) In “Volcano Skate”, the player has to perform skating movements in order to skate up a volcano. Once the player reaches the top, he has to skate downhill and jump over upcoming barriers. (6) In “Slalom Grove”, the player again has to imitate sequences of movements (comparable to aerobics) related to slalom skiing. In each activity, a higher score can be achieved through more movement and by performing the movements more precisely.

Participants were instructed to play for a duration of 15 minutes at moderate to vigorous intensities. Although, no study has investigated a dose-response relationship in children with ADHD or healthy children, a duration of app. 15 minutes at moderate to vigorous intensity was chosen for the current study. This decision was based on a previous meta-analysis^33^, which recommends durations of 11–20 minutes at this intensity, as well as a study on healthy adolescents that found positive effects on EFs after about 15 minutes of exergaming^50^.

In the *Control* condition, the participants watched a documentary report about mountain running, with a similar duration to the *Exergaming* condition.

### Manipulation check

To test whether the experimental manipulation had succeeded, several subjective and objective measures were applied. The *OMNI scale of perceived exertion* was used as a subjective measure of physical exertion. Evidence for concurrent and construct validity has been demonstrated^51^. The heart rate (beats per minute) was recorded using the Polar Team 2 Pro system (Polar Electro Oy, Kempele, Finland) as an objective measure. Children’s maximal heart rate was predicted using the formula 208–0.7*age^52^. The actual and the predicted heart rates were used to calculate the exercise intensity. Moderate to vigorous intensity was defined as 55–90% of the maximal heart rate^53^. To measure cognitive engagement, the *Self-Assessment Manikin* was adapted to ask about the perceived cognitive engagement of the activity. Even though it has not been validated, this instrument has been proven to be feasible in studies with children and adolescents^54^. Valence and arousal were measured using the Self-Assessment Manikin. The Self-Assessment Manikin is a widely used, non-verbal, pictorial, one-item assessment to measure a person’s affective reaction to stimuli. Acceptable psychometric properties have been demonstrated^55^. In addition, *enjoyment* was measured using three self-developed questions: (1) “How much did you like the activity?” (2) “Did you feel comfortable doing the activity?” (3) “Did you enjoy doing the activity?”. The questions had to be answered on a 4-point Likert scale (1–4 points for each item), and the sum was calculated as the enjoyment score. The internal consistency for the three questions was acceptable (Cronbach’s alpha = 0.81).

### EFs

In counterbalanced order, EFs were assessed by means of two computer tasks using E-Prime Software (Psychology Software Tools, Pittsburgh, PA). The two tasks took about 15 minutes to complete. These tasks have been proven to be reliable and valid measures of EFs for healthy children and adolescents^56–58^. In the present sample, the retest reliability of pre- and post-test reaction times for inhibition (*r* = 0.761), switching (*r* = 0.772), global switch costs (*r* = 0.715) and visual working memory (*r* = 0.679) were acceptable, in particular considering that an intervention took place between the two measurements which could potentially affect reaction times.

In order to minimize confounding effects and to avoid children being overtaxed by the cognitive testing, it had to be as brief as possible. Therefore, *inhibition* and *switching* were included in one task – a single modified Flanker Task^59–61^. The Flanker Task is widely used in children with ADHD. Acceptable psychometric properties have been demonstrated for healthy schoolchildren^62^. In this task, five fish were depicted on a screen. These fish could be red or yellow and children were instructed to feed fish by pressing an external response button. The button pressed indicated the direction in which mouth of the fish was pointing. The task consisted of two blocks: In the first block, the target fish was situated in the middle (red fish), flanking fish could either swim in the same direction as the target fish (congruent trials) or in the opposite direction (inhibition trials). This block consisted of 40 trials, including 20 congruent and 20 incongruent trials in a randomized order. The second block also consisted of 40 trials in total, including 20 congruent and 20 incongruent trials (inhibition trials). In 20 of these trials, the fish were red and in 20 trials the fish were yellow. For the red fish, the fish in the middle was the target fish; for the yellow fish, it was the four flanking fish. Children had to adapt their response depending on the color of the stimuli and either feed the fish in the middle (red fish) or the flanking fish (yellow fish). A switch between the two rules was required in 20 trials, when the color of the stimuli changed (switching trials). Mean reaction times and accuracy of the congruent and incongruent trials (1^st^ and 2^nd^ block) as well as the switching trials (2^nd^ block) were calculated. In addition, the global switch costs were calculated^63^. Since trials in the 2^nd^ block not only require children to switch between different tasks, but also contain inhibitory demands, the difference between the reaction times of the 2^nd^ block and the 1^st^ block was calculated to control for the inhibition component. To ensure that the participants understood the task correctly, they completed five practice trials before each block. If their performance was below 60% they had an additional practice loop including another five practice trials. The inter-stimuli intervals were varied randomly between 800 to 1400 ms.

*Visual working memory* was assessed using a modified version of the Color Span Backwards Task^64,65^. This has been shown to be valid, and an acceptable retest reliability has been demonstrated in children^64^. In this task, coins of different colors appear on the screen one after another, embedded in a cover story about a dwarf. The children have to remember the color of the coins and repeat their appearance in reverse order. After a short explanation and three training trials, the first six trials started. They consisted of two coins each and if the child answered three or more trials correctly, the span was increased by one coin for the next six trials. The span was increased until the child made more than three mistakes. In this case, the test was terminated after conducting the remaining trials for the current span. At the end, the sum of correct responses was counted as the outcome score.

### Statistical analyses

Statistical tests were performed using SPSS 23.0 (SPSS Inc., Chicago, IL, USA). In the *outlier analysis*, trials with a reaction time below 150 ms were excluded as anticipatory (interindividual outliers, Flanker: 0.2%). In a next step, trials with reaction times deviating by more than 3 *SD* from the child’s mean (intraindividual outliers, Flanker: 1.6%) were excluded as well. Only correct trials were included in the calculation of reaction times.

*Preliminary analyses* were performed using *t*-tests for between-group comparisons of demographic variables, detecting no significant differences (see Table 1). In addition, Pearson correlations were calculated between the pre-test values of the dependent variables (reaction times), in order to investigate whether EFs are intercorrelated. For the *main analyses*, analyses of covariance (ANCOVAs) using pre-test performance as covariates and post-test performance as the dependent variable were conducted, in order to increase statistical power and reduce potential bias due to baseline imbalances^66,67^.

Partial eta square (η^2^_p_) was reported as an estimate of effect size; the magnitude of η^2^_p_ was interpreted using benchmarks suggested by Cohen^68^, distinguishing between small (~0.0099), moderate (~0.0588), and large (~0.1379) effect sizes. The level of significance was set at *p* < 0.05 for all analyses.

## Results

### Manipulation check and preliminary analyses

As expected, children’s heart rates (*t*(44) = 18.32, *p* < 0.0005, η^2^_*p*_ = 0.884) and perceived physical exertion (*t*(44) = 8.48, *p* < 0.0005, η^2^_*p*_ = 0.620) were significantly increased (Table 2). All children participating in the *Exergaming* group were in the moderate to vigorous intensity range for at least 14 minutes (*M* = 14.59, *SD* = 0.50) and the average exercise intensity ranged between 64.95% and 78.66% (*SD* = 3.71) of the maximal heart rate. Moreover, children in the *Exergaming* group were more cognitively challenged (*t*(44) = 3.18, *p* = 0.003, η^2^_*p*_ = 0.195) and aroused (*t*(44) = 2.78, *p* = 0.008, η^2^_*p*_ = 0.150) than the ones in the *Control* group. However, both groups showed a comparably high level of enjoyment (*t*(44) = 1.93, *p* = 0.061, η^2^_*p*_ = 0.078) and valence (*t*(44) = 0.16, *p* = 0.873, η^2^_*p*_ = 0.001). These results indicate a successful experimental manipulation. In addition, correlational analyses showed that core EFs and particularly inhibition and switching are interrelated (see Table 3). Looking at reaction times in inhibition and global switch costs, the interrelation is reduced (*r* = 0.562) because this score controls for the inhibition component. Nevertheless, the three core EFs seem to share variance.

**Table 2:** Descriptive statistics and between-group analyses of manipulation check.

	Exergaming (*n* = 24)	Control (*n* = 22)	η^2^_p_
	*M* (*SD*)	*M* (*SD*)	
Heart rate (beats/min)	147.73 (7.26)*	83.68 (6.09)	0.960
OMNI scale	5.92 (2.15)*	1.32 (1.43)	0.620
Enjoyment rating	11.10 (1.91)	10.09 (1.63)	0.078
SAM			
Valence	7.75 (1.68)	7.68 (1.13)	0.001
Arousal	6.50 (2.19)*	4.86 (1.75)	0.150
Cognitive engagement	5.67 (2.41)*	3.59 (1.84)	0.195

Note. OMNI scale: Rating of Perceived Exertion. SAM: Self-Assessment-Manikin.

Significant differences between the two groups are indicated by an asterisk (*p < 0.05).

**Table 3:** Correlational matrix showing the relationships between executive function performances (reaction times).

	Inhibition	Switching	Global switch costs
Inhibition	1		
Switching	0.829*	1	
Global switch costs	0.562*	0.756*	1
Working memory	−0.312*	−0.338*	−0.201

Note. *Correlation is significant at the *p* < 0.05 level.

### EFs

The detailed descriptive statistics of EF performance, including effect sizes for ANCOVA comparisons, are presented in Table 4. Regarding the reaction times, a trend in favor of the *Exergaming* group was revealed in congruent trials of the Flanker Task (*F*_Flanker_(2, 43) = 3.387, *p* = 0.073, η^2^_*p*_ = 0.073). In addition, the *Exergaming* group displayed shorter reaction times in the Flanker Task in incongruent (*F*_Flanker_(2, 43) = 5.69, *p* = 0.022, η^2^_*p*_ = 0.117) and switching trials (*F*_Flanker_(2, 43) = 5.50, *p* = 0.024, η^2^_*p*_ = 0.113), as well as global switch costs (*F*_Flanker_(2, 43) = 4.45, *p* = 0.041, η^2^_*p*_ = 0.094). The effects observed were moderate, with the largest effects for inhibition (η^2^_*p*_ = 0.117).

**Table 4:** Descriptive statistics of executive function performance and effect sizes for ANCOVA comparisons.

	Exergaming (*n* = 24)		Control (*n* = 22)		η^2^_p_
	Pre-test *M* (*SD*)	Post-test *M* (*SD*)	Pre-test *M* (*SD*)	Post-test *M* (*SD*)	
Flanker Task reaction times (in milliseconds)					
Congruent (ms)	763 (201)	700 (156)	801 (256)	798 (20)	0.073
Inhibition (ms)	981 (263)	842 (162)*	1013 (319)	959 (266)	0.117
Switching (ms)	1031 (276)	909 (200)*	1083 (303)	1066 (327)	0.113
Global switch costs (ms)	424 (218)	324 (177)*	559 (249)	495 (214)	0.094
Flanker Task accuracy (% of correct responses)					
Congruent (%)	97	98	97	99	0.046
Inhibition (%)	90	89	90	90	0.002
Switching (%)	90	90	91	90	0.006
Color span backwards (sum of correct responses)					
Working memory	14 (2.84)	14 (3.84)	14 (4.14)	14 (3.71)	0.000

Note. ms: milliseconds. % percentage of correct responses. Significant differences in post-test between two groups with adjusting pre-test are indicated by an asterisk (*p < 0.05).

Regarding accuracy scores of the Flanker Task, no significant differences were revealed for congruent (*F*_Flanker_(2, 43) = 2.01, *p* = 0.157, η^2^_*p*_ = 0.046), incongruent (*F*_Flanker_(2, 43) = 0.09, *p* = 0.770, η^2^_*p*_ = 0.002) or for switching trials (*F*_Flanker_(2, 43) = 0.26, *p* = 0.616, η^2^_*p*_ = 0.006).

For visual working memory performance, no significant difference was revealed between the two groups in the Color Span Backwards Task (*F*_Color span_(2, 43) = 0.00, *p* = 0.995, η^2^_*p*_ = 0.013).

## Discussion

The present study utilized an exergaming intervention, considering all three core EFs in one sample (inhibition, switching and visual working memory), to investigate the effects of acute physical activity on EFs in children with ADHD. The results revealed that completing an acute exergaming intervention of a moderate to vigorous intensity for at least 14 min had significant beneficial effects on reaction times in inhibition and switching, but not on accuracy or visual working memory performance. Thus, the type of core EFs and potentially the way these are measured play a mediating role between acute physical activity and cognitive function.

The positive effect of acute physical activity on inhibition reaction times corresponds to that in previous studies which examined the relationship between acute exercise interventions and EFs (inhibition) among children with ADHD. It should be noted that in these studies, inhibition was assessed using a variety of cognitive tasks including Stroop^36,39^, Go/No-Go^38,40^, Eriksen Flanker^37^ and the Continuous Performance Test^69^, mostly finding moderate effect sizes. Considering that in the present study an effect was detected using the Flanker Task, it could be argued that the benefits of acute exercise for inhibition performance are evident regardless of the task utilized to measure it. Moreover, finding the largest effect sizes for inhibition (η^2^_*p*_ = 0.117), might reflect a robust effect of acute exercise on inhibition. To explain these improvements, neuroelectric adjustments have been proposed as the underlying mechanism, since alterations to neuroelectric activation have been shown to accompany increases in EF performance^37,38,70^. These findings indicate enhanced regulatory processes^37^ and more efficient response preparation^38^. Another potential mechanism might be an increased functional connectivity between networks related to attention and executive control following acute physical activity^29^. Taken together, although the exact underlying mechanisms are still not fully understood, the behavioral data from this study, along with previous studies, indicates that in particular the inhibition component, when measured by means of the reaction time, benefits from acute physical activity in children with ADHD.

In terms of switching, our study revealed significant improvements in reaction times following the acute exergaming intervention. To our knowledge, only three studies have examined acute physical activity on switching performance in children with ADHD. These studies produced contradictory results, despite utilizing a similar physical activity protocol in terms of exercise intensity, duration and qualitative characteristics. Two studies detected positive effects (moderate to large effect sizes) on either switching indexes of the Wisconsin Card Sorting Test^36^, or global switch costs in a task-switching paradigm^70^, whereas another study failed to find significant effects on switching performance assessed using the Trail Making Task^39^. These contrasting results suggest that the effects of acute physical activity on switching may not be as strong as that observed for inhibition, which is supported in this present study, in which the effects on switching reaction times (η^2^_*p*_ = 0.113) and global switch costs (η^2^_*p*_ = 0.094) were found to be smaller than that on inhibition (η^2^_*p*_ = 0.117). Notably, Piepmeier *et al*.^39^ included only 14 children with ADHD, and therefore the sample size (*n* = 14) may have been too small to detect this effect. Another difference of the current study is that cognitively engaging physical activity was used. This physical activity consisted of movements which had to be learned, for example, or of quick reactions to different types of stimuli. Since both quantitative and qualitative characteristics seem to enhance cognitive processing^71^, the conflicting results could be attributed to both the qualitative physical activity characteristics and to the cognitive task used. This study gives further evidence in favor of the positive effects of acute physical activity on switching performance using the modified Flanker Task in children with ADHD, warranting a further examination of these effects.

Although this study revealed no significant effect on visual working memory, it seems to agree with the existing literature. Previous studies of similar design using working memory tasks as outcome variables in children with ADHD, have also found no effect^41,42^. Moreover, a similar result was obtained in college students with ADHD, i.e. beneficial effects on inhibition (Stroop task completion times) but not on working memory performance^72^. In contrast, Hung *et al*.^70^ utilized a task-switching paradigm, claiming that improvements in switching are closely connected to working memory performance. They also detected beneficial effects on response times of switching and an enhanced P3 amplitude (EEG) which, according to them, reflects enhanced working memory processes. Although these findings seem contradictory at first glance, they might be explained by the demands of the working memory tasks themselves. In fact, the working memory tasks used in both previous studies and this present study did not consider response speed as an outcome. It seems that working memory accuracy is affected less by acute physical activity in children with ADHD. However tasks including response speed and working memory performance seem to be more prone to the effects of acute physical activity^73^.

Similarly to visual working memory accuracy, no effects on the accuracy scores of inhibition and switching were found. This finding is in line with the majority of studies involving children with ADHD^38,39,69,70^. In addition, a meta-analysis indicates that the positive effects of acute, intermediate-intensity exercise are positively associated with response speed in working memory tasks, however detrimental for accuracy^73^. The authors^73^ indicate that increased catecholamine concentrations in the brain might have different sensitivities for speed of processing and for accuracy^73^. Considering that acute exercise seems to lead to an exercise-induced release in catecholamines^29^ this explanation seems conceivable. However, to our knowledge at least three studies did find positive effects in children with ADHD^23,37,40^. Therefore, an extended explanation of the diverging results might be found in speed-accuracy tradeoffs^74^. One could speculate that children are able to invest the additional attentional resources resulting from acute exercise either in response speed or in accuracy. Since a ceiling effect was evident for response accuracy in the current study, additional attentional resources could only be allocated to response speed. Future experimental studies should systematically investigate the effects of physical activity on speed-accuracy tradeoffs, in particular, whether children (with ADHD) are able to allocate acute exercise benefits to either accuracy or reaction times.

This study applied a physical activity condition of moderate to vigorous intensity lasting 14–15 minutes. Although the majority of studies typically used a 30-minute protocol, beneficial effects of physical activity, performed at moderate to vigorous intensities, have been shown in children with ADHD ranging from 5^40^ to 30 minutes^36^. Therefore, the beneficial effects of acute physical activity on EFs in this study seem to be in line with the empirical evidence, indicating that in children with ADHD an acute physical activity of 14–15 minutes at moderate to vigorous intensity is sufficient to elicit positive effects on EFs. Chang *et al*.^75^ compared interventions lasting 10 min, 20 min and 45 min, with a no exercise control condition, and found that the 20 min condition exhibited the highest EF performance for adults. Thus, a potential explanation for the missing effects on accuracy measures and visual working memory might be that the duration of the physical activity was too short. In contrast, since other studies applying cognitively engaging moderate intensity physical activity in children have found positive effects even for durations of 10 min^43,76^, one might speculate that shorter durations are able to evoke positive effects on cognitive performance in children and that the type of physical activity influences these effects^76^. This notion is supported by a recent study showing that cognitively engaging activities (with and without physical activity) can have a positive impact on EFs^43^. Therefore, one could speculate that cognitive engagement leads to increased arousal. This increased arousal in turn might compensate for shorter durations or lower intensities. This is relevant when considering physical activity breaks at school, where shorter durations seem advantageous as they can be conducted during the recess session. Therefore, future studies are needed to investigate a) the dose-response relationship of intensity and duration in children and b) the type of physical activity, by systematically varying the three variables mentioned.

One major novelty of the current study is the use of exergaming as the physical activity modality. Previous studies have exclusively applied aerobic exercises, such as treadmill running^36–38,40^ or a cycling ergometer^39^. The exergame used consisted of an additional cognitively challenging activity, including non-automated movements making high demands on coordination and speed of action. Exergaming was investigated based on the theoretical assumption that cognitively challenging physical activity, such as coordinative exercise, pre-activates brain regions used to control higher-order cognitive processes, thus leading to better performance^77–80^. Indeed, a cognitively challenging exergame has been shown to produce larger effects on EFs in adolescents than a version of the game with a low level of cognitively challenge^50^. Against the background of existing deficits in motor abilities in children with ADHD, and the important association between motor abilities, EFs and academic achievement^81^, the results of this current study are highly relevant. They indicate that using a physical activity intervention that focuses on both learning and motor skill development has the potential to improve EFs in children with ADHD. Furthermore, results from the subjective ratings revealed that children with ADHD enjoyed playing the exergame to the same extent as watching videos in the control group. Considering that children with ADHD frequently find conventional training programs tiring and boring, due to motivational deficits and a greater need for direct rewards^82^, the use of alternative exercise modalities that considers motivation for this special population is encouraged.

There are certain limitations to the present study. First, the sample size and associated statistical power was not large enough to consider any sub-group analyses (e.g., ADHD symptoms, medication dosage, BMI, gender). Although the study participants seem to be representative for children with ADHD in this age group (with regard to background variables such as pubertal and socioeconomic status), sub-group analyses would give further insight into the interplay of these variables. Therefore, these potential mediators could be an important avenue for future studies. Second, most of the participants were currently being treated with medication; therefore the possible effects on medication-naïve patients and whether physical activity might be an alternative to medication remain unclear. Future research should therefore explore the role of physical activity in the absence of medication, helping to isolate the origins of these benefits. Third, we included 82.6% boys in the current study. This represents a slightly higher rate compared with the prevalence in population-based studies^1^. However, when considering clinical samples, the male-to-female ratio was within the normal range^3^. Because we recruited our participants through an association for parents and caregivers of children with ADHD, it is likely that this study consists of a clinical sample. However, we cannot rule out the possibility that male participants were particularly attracted by our study because of the intervention itself. Although this study included a larger number of female participants, compared to most previous studies on acute exercise in children with ADHD^36,38,40–42,69,70^, it nevertheless remains unclear whether the results obtained are also true for a majority of girls associated with ADHD. Fourth, the current study did not have the power to investigate the factorial structure of EFs in children with ADHD. We tried to assess the three core EFs separately, because the current study was built upon the theoretical model of Miyake^13^. However, the correlational analyses show that inhibition and global switch costs are interrelated (*r* = 0.562). Therefore, it remains unclear whether inhibition and switching are two different constructs. However, a potential explanation for this finding might be the so-called task impurity problem. Accordingly, the three core EFs can hardly be assessed in isolation, because an EF assessment always has to be embedded in a certain task context, which might also trigger other EFs^83^. Since studies on the exact factor structure of the EFs are still dissenting regarding how many factors (1, 2 or 3 factors) are evident in children^84^, future studies should further investigate the factorial structure of EFs in healthy children and in children with ADHD. Fifth, in the current study, moderate to vigorous intensity was estimated using the formula 208–07*age^52^. We are aware that this is a rough estimate of maximal heart rate; however unfortunately we did not assess resting and maximal heart rate in the current study. Referring to the cut-offs provided by Norten, Norten & Sadgrove^53^, when looking at the average heart rate during acute physical activity, we are tempted to believe that the intervention was of moderate to vigorous intensity. Finally, only a cognitively challenging physical activity was used in the experimental condition, and we are therefore unable to distinguish whether the cognitive or the physical challenge was responsible for improvements in EF performance. Further work is required using a two-way experimental design in order to investigate the underlying mechanisms^43,85^.

## Conclusion

The current study expands upon existing findings by measuring the effects of acute physical activity using exergaming on all three core EFs in a single study. Reaction times in inhibition and switching, but not accuracy or visual working memory, were enhanced following the acute physical activity. Our results suggest that acute physical activity might have specific effects on EFs (in this case on reaction times in inhibition and switching) in children with ADHD. Moreover, acute cognitively engaging exergaming with moderate to vigorous intensity for 14–15 min might serve as a promising tool to increase physical activity levels and enhance EFs in the future. Our study warrants further research investigating the effects of exergaming and acute physical activity on all three core EFs, taking into consideration the present limitations and suggestions made.
